# Assessing the Anti-Cryptococcus Antifungal Potential of Artemisinin

**DOI:** 10.3390/ijms26209953

**Published:** 2025-10-13

**Authors:** Maphori Maliehe, Jacobus Albertyn, Olihile M. Sebolai

**Affiliations:** Department of Microbiology and Biochemistry; University of the Free State, Bloemfontein 9301, South Africa; maphorimaliehe1@gmail.com (M.M.); albertynj@ufs.ac.za (J.A.)

**Keywords:** antifungal, Artemisinin (ART), *Cryptococcus* (*C.*) *neoformans*, drug repurposing, *Galleria* (*G.*) *mellonella*, insect antimicrobial response

## Abstract

*Cryptococcus neoformans* (*C. neoformans*) has emerged as a global pathogen of concern. While much is known about its pathobiology, its management is complicated by strains displaying non-fluconazole susceptibility. This contribution assessed the repurposing of artemisinin (ART) as an anti-*Cryptococcus* antifungal. An in vitro susceptibility assay was performed to assess the drug response of cells. To establish the ART mode of action, assays examining mitochondrial health were set up. The phagocytosis efficiency of a murine macrophage cell line towards ART-treated and non-treated cells was determined. To complement this, the immunomodulatory effects of ART were further characterised in *Galleria mellonella* (*G. mellonella*) by assessing haemocytes’ phagocytosis and expression of immune genes, *i.e*., insect metalloproteinase inhibitor (IMPI) and hemolin, essential for the insect antimicrobial response. In the end, the survival rate of infected larvae was calculated. We established that ART was antifungal, with cell death triggered by the uncoupling of the cytochrome *c* (cyt *c*) from the mitochondria, leading to activation of caspase-3-dependent-like apoptosis. Moreover, treatment induced ultrastructural changes with treated cells appearing more deformed than non-treated cells (*p* < 0.05). Treatment also increased the susceptibility of cells towards both macrophage and haemocyte phagocytosis compared to non-treated cells (*p* < 0.05). Importantly, treatment seemed to weaken the cells, decreasing their virulence potential based on analysis of the expression of the immune gene markers, which translated into treatment rescuing 75% of the larvae infected with 0.1 ART-treated cells. These preliminary findings support the repurposing of ART as an anti-*Cryptococcus* antifungal.

## 1. Introduction

*Cryptococcus neoformans* (*C. neoformans*) is an encapsulated opportunistic fungal pathogen responsible for life-threatening infections such as cryptococcal meningitis, especially in individuals with advanced HIV disease [1]. Therefore, its inclusion in the first WHO Fungal Priority Pathogens List signifies its appreciation as a global pathogen of medical importance [2]. While we have known about cryptococcal pathogenesis for over 130 years, its associated mortality remains particularly high in endemic regions [3,4]. In part, this is because of strains exhibiting non-fluconazole susceptibility [5,6]. To compound matters, amphotericin B, which is the gold standard for managing cryptococcal infections, induces severe side effects in patients [7]. Due to these reasons, there is a need to find alternative treatment options, and drug repurposing may be the solution.

Drug repurposing is an attractive approach, as the safety profile of the concerned drug is already known, and this helps overcome the cost and time constraints associated with establishing a new antifungal agent [8]. This approach has led to uncovering that auranofin, a disease-modifying antirheumatic drug, exhibits in vitro antifungal activity against *C. neoformans* and other fungi by inducing mitochondrial dysfunction via Mia40-Erv1 inhibition and interfering with the thioredoxin pathway, leading to oxidative stress [9,10,11]. Importantly, the Thangamani et al. study suggested that the lipophilic nature of this drug allowed it to enter cells and affect the fungal stress response, thus contributing to the reported efficacy [9]. Therefore, by comparing the effects of diverse compounds, we may gain insight into which molecular features, e.g., lipophilicity, are important for antifungal success, which can help guide other studies to improve their outcomes. 

In this study, the concerned drug was the lipophilic artemisinin (ART). Traditionally, this drug is indicated for controlling *Plasmodium*, the aetiological agent of malaria [12]. The compound is reported to inactivate *Plasmodium* by targeting its mitochondria [13]. Of interest, this drug has previously been shown to control microbes in in vitro studies. Moreover, Galal et al. demonstrated that several ART derivatives exhibit potent activity against *C. neoformans* and *Candida albicans* [14], while recent work by Zhu et al. demonstrated that ART could modulate fungal membrane integrity through elevating the ergosterol levels in *Candida albicans*, and in turn, synergised with amphotericin B to enhance its antifungal efficacy [15], and Sumlu et al. reported that it suppresses key biofilm-associated genes, thus disrupting biofilm formation [16]. Thus, the current work aims to build on this existing antifungal literature. As cryptococcal cells are strict aerobes, they rely on functional mitochondria for cellular energy generation [17]; thus, we rationalise that a drug that may impair the cryptococcal mitochondria would result in deleterious effects.

When aiming to repurpose a drug, its success should not be limited to in vitro efficacy, but its utility should extend to impacting the host–pathogen interactions and modulating the immune response in favour of the host. The latter is of particular importance for infections involving *C. neoformans*. This is because cryptococcal cells possess an arsenal of microbial factors, such as the polysaccharide capsule, which they can deploy to impair phagocytosis and modulate the immune response [18,19]. Unfortunately, this in part allows for cells to hide inside phagocytic cells in a Trojan horse-like manner, thus escaping the mononuclear phagocytic system and disseminating to reach distal organ systems. To this end, we had set up in vitro tests to evaluate the effects of ART and artesunate (ARS), a semi-synthetic derivative of ART, which was included for its improved solubility over ART, on the growth of cells. ART was further used to assess its impact on the mitochondrial health of cryptococcal cells. This was complemented by examining how ART treatment could resolve a systemic cryptococcal infection in *Galleria mellonella* (*G. mellonella*). Thus, the designed experiments represented initial steps in a pre-clinical translational programme that may help to highlight the clinical relevance of administering ART.

## 2. Results

### 2.1. Artemisinin Displays Anti-Cryptococcus Activity

**Figure 1:**
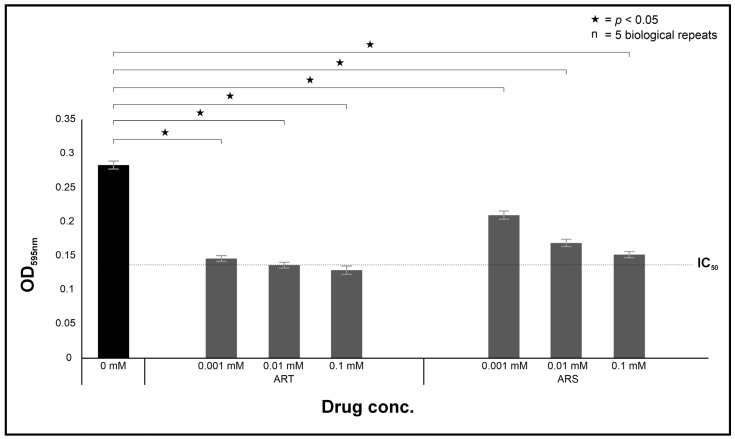
The in vitro effect of artemisinin (ART) and artesunate (ARS) on the growth of *C. neoformans* H99 cells relative to the growth of non-treated cells. “n” = number of biological repeats.

The in vitro susceptibility results of cryptococcal cells towards ART and ARS, which is structurally related to ART, are summarised in Figure 1. In this part of the study, the half maximal inhibitory concentration (IC_50_) was used to determine the potency of the tested drugs in inhibiting the in vitro growth of cells by 50% relative to non-treated cells. As the two drugs were prepared to the same molar strength, it was possible to deduce that ART was more potent than ARS (*p* < 0.0001), when directly comparing the cells’ response to the two drugs, and importantly, at a concentration of 0.1 mM, ART could inhibit growth by more than 50% while this was not the case for ARS. For this reason, the subsequent studies were limited to the use of ART. Interestingly, in their work, Galal et al. [14] showed that at a concentration of 0.003 mM, anhydrodihydroartemisinin could reduce growth by 50%. This suggests that, when compared to our current data, this structural modification may be more potent [14].

**Figure 2:**
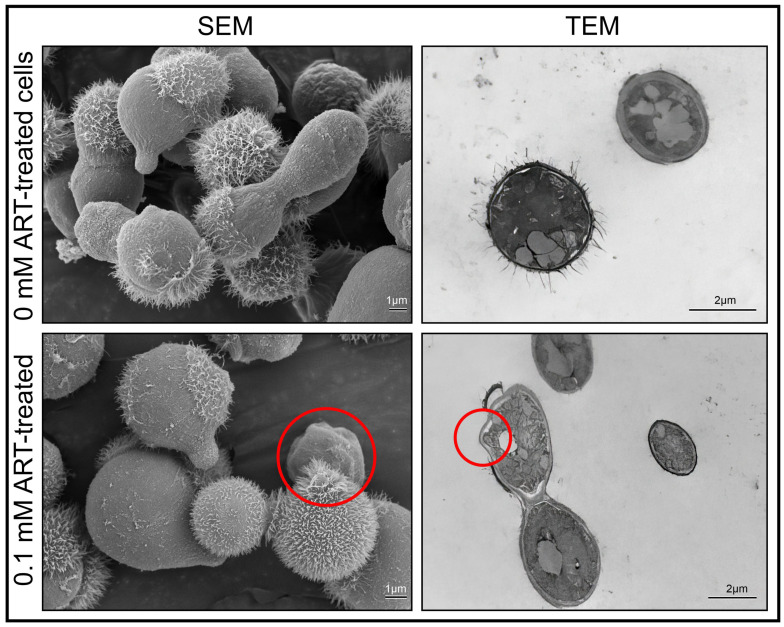
The collated electron microscopy images showing the morphological differences of cryptococcal cells’ ultrastructure following artemisinin (ART) treatment. The circles highlight the ultrastructural defects, noted as indentations, on the cell’s surface induced by treatment. SEM = scanning electron microscopy, TEM = transmission electron microscopy.

Furthermore, the captured SEM and TEM micrographs, depicting representative cells obtained from the different experimental conditions, i.e., 0 mM ART and 0.1 mM ART, were collated into Figure 2 to help make deductions regarding the effects of drug exposure. The figure showed 0.1 mM treated cells with an altered morphology (note the red circles) in the form of collapsed cell walls, referred to herein as “indentations”. 

**Figure 3:**
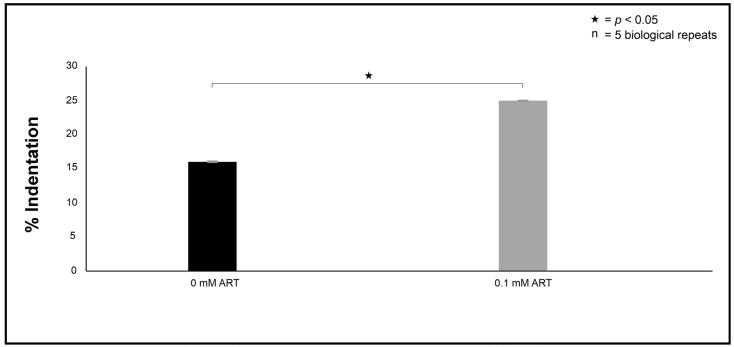
The quantification of indentations observed on the cell’s ultrastructure. The data was calculated using a random sample of 100 cells that were only examined with TEM. “n” = number of biological repeats.

While these indentations may be incidental due to sample preparation, they were observed to be more associated with the treated cells than the non-treated cells, as the respective % indentation is shown in Figure 3.

### 2.2. Artemisinin Treatment Impairs Mitochondrial Function

**Figure 4:**
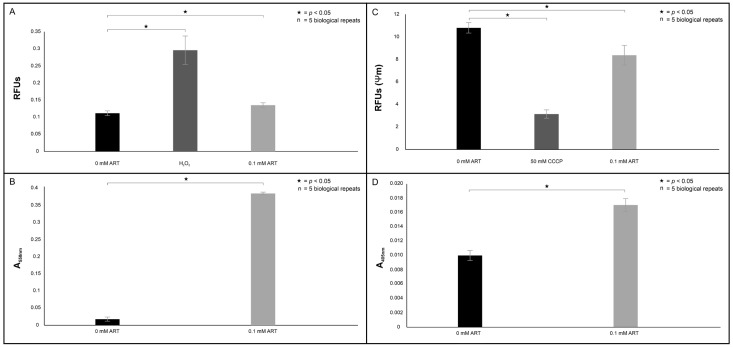
The collated image shows the effects of artemisinin (ART) treatment on the mitochondrial health of cryptococcal cells and initiation of apoptosis. (**A**) depicts the accumulation of intracellular reactive oxygen species (ROS) in treated cells relative to non-treated cells, whereas (**B)** shows the accumulation of cyt *c* in the cytoplasm, which is indicative of its uncoupling from the mitochondrial electron transport chain. (**C**) provides evidence of a dysfunctional mitochondrial membrane characterised by loss of membrane potential in treated cells relative to non-treated cells, while (**D**) summarises caspase3-like activation in treated cells relative to non-treated cells. RFUs = relative fluorescence units, Ψm = mitochondrial membrane potential. “n” = number of biological repeats.

The ROS accumulation data are summarised in Figure 4A. Treatment of cells with 0.1 mM ART resulted in significant detection of ROS compared to non-treated cells (*p* = 0.0431). The generated ROS may be a result of the uncoupling of the electron transport chain. This is because cyt *c* is essential for shuttling electrons between complex III and complex IV to enable the complete reduction of oxygen to water. Thus, when dislodged, electrons accumulate in upstream complexes, resulting in incomplete reduction of oxygen and the generation of ROS. To confirm the latter, we determined if cyt *c* was dislodged from the mitochondria. The results showing the accumulation of the dislodged cyt *c* in the cytoplasm are summarised in Figure 4B. Here, it was determined that cells treated with 0.1 mM ART accumulated significantly more cyt *c* in the cytoplasm relative to non-treated cells (*p* = 0.0003). One of the cardinal indicators of mitochondrial dysfunction is loss of membrane potential. The effects of treatment on membrane potential are summarised in Figure 4C. As expected, CCCP, a known inhibitor of oxidative phosphorylation, induced a significant loss of membrane potential compared to non-treated cells (*p* = 0.002). Likewise, treatment of cells with 0.1 mM ART resulted in a significant loss of membrane potential compared to non-treated cells (*p* = 0.05). 

**Figure 5:**
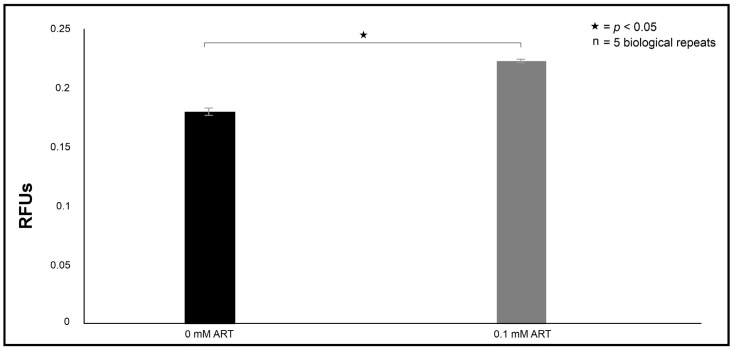
The effect of artemisinin (ART) treatment on the selective permeability of cell membranes relative to the permeability of non-treated cells. The propidium iodide (PI) stain was used to assess intracellular accumulation following drug treatment. RFUs = relative fluorescence units. “n” = number of biological repeats.

The latter is supported by the evidence of loss of membrane selective permeability as treated cells significantly accumulated the PI stain compared to non-treated cells (*p* = 0.0001) (Figure 5). When reacted with this stain, healthy cells can exclude the stain due to an intact membrane, whereas cells with damaged or permeable membranes allow the stain to intercalate with their DNA and RNA to emit fluorescence, an indication of a compromised cytoplasmic membrane [20]. This loss of selective permeability suggests it is foreseeable for the uncoupled cyt *c* to leak through the now dysfunctional membrane to localise in the cytoplasm to trigger caspase-induced apoptosis. The data summarising the activation of caspase 3 within the 0 mM ART and 0.1 mM ART treated cells are shown in Figure 4D. Here, we determined that caspase 3 activation was significantly more pronounced in 0.1 mM ART-treated cells compared to non-treated cells (*p* = 0.032).

### 2.3. Artemisinin Treatment Enhances Cryptococcal Susceptibility Towards Macrophage Phagocytosis

**Figure 6:**
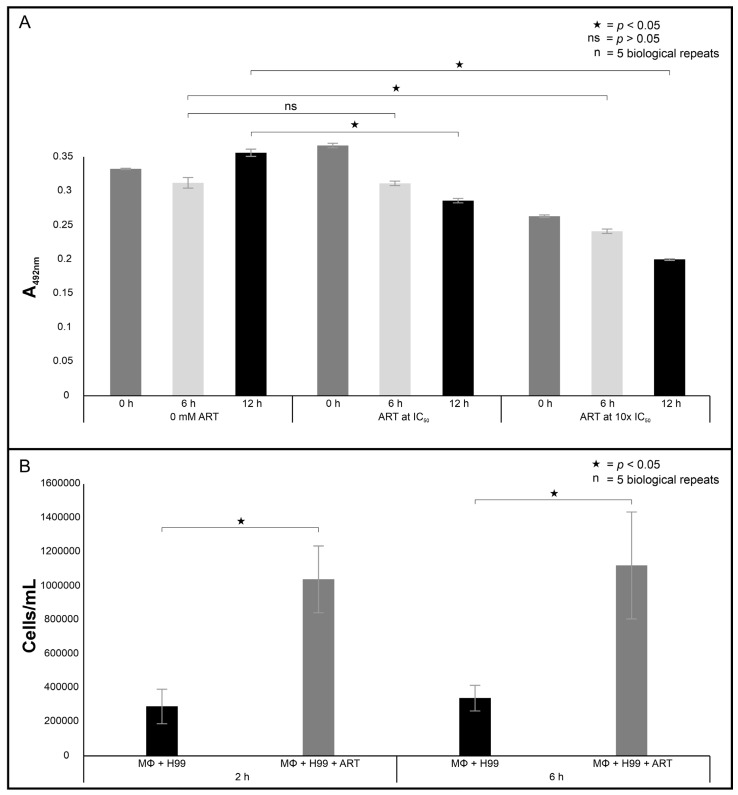
The in vitro effect of artemisinin (ART) on macrophages. (**A**) depicts the health of macrophages following ART treatment as measured following a tetrazolium reaction, while (**B**) shows ART chemosensitising quality as measured by quantifying macrophage efficiency to phagocytose internalised cryptococcal cells. H99 = *C. neoformans* H99 cells, MΦ = macrophages. “n” = number of biological repeats.

It was first determined if ART treatment affected the vitality of macrophages using the XTT assay. The obtained results are summarised in Figure 6A. It was determined that ART at 0.1 mM (IC_50_) reduced the metabolic activity by 2% (*p* = 0.814) after 6 h of ART exposure and by 14% (*p* < 0.0001) at 12 h of ART exposure, while at 1 mM (10x the IC_50_) it reduced the metabolic activity by 28% (*p* < 0.0001) after 6 h of ART exposure and by 41% (*p* < 0.0001) after 12 h of ART exposure, when compared to non-treated cells. The above suggests the short-term poisoning potential of ART was not detrimental to reduce the metabolic activity by more than 50%. Importantly, concerning the phagocytosis results, it was noted that cells that were treated with 0.1 mM ART were significantly more susceptible to macrophage phagocytosis than non-treated cells at both 2 h (*p* = 0.03) and 6 h (*p* = 0.038) time intervals (Figure 6B).

### 2.4. Artemisinin Treatment Increases the Survival Rate of the Infected Larvae

**Figure 7:**
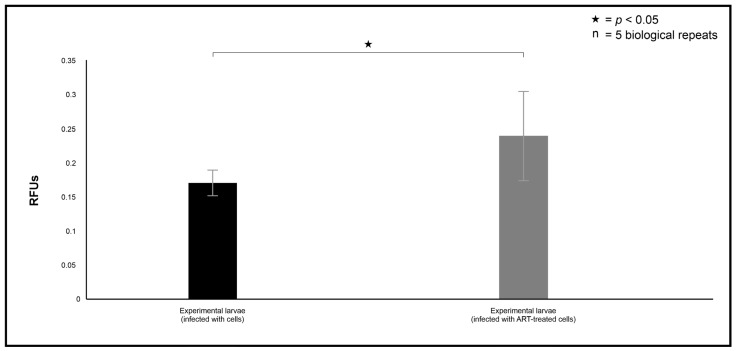
The quantification of the *Galleria mellonella* haemocytes’ efficiency to phagocytose the artemisinin (ART)-treated cryptococcal cells relative to the non-treated cells. The pHrodo stain was used as the phagocytosis stain. RFUs = relative fluorescence units. “n” = number of biological repeats.

The ability of cryptococcal cells to display increased phagocytosis susceptibility was not limited to macrophages but was also observed in the haemocytes (Figure 7). The haemocytes were noted to significantly phagocytose more ART-treated cells 24 h post-infection than 0 mM ART-treated cells (*p* = 0.04). This data was in line with the macrophage phagocytosis results (Figure 6B) and, thus, corroborated the efficacy of ART as a compound that can chemosensitise phagocytic cells, in turn increasing their ability to resolve internalised cells.

**Figure 8:**
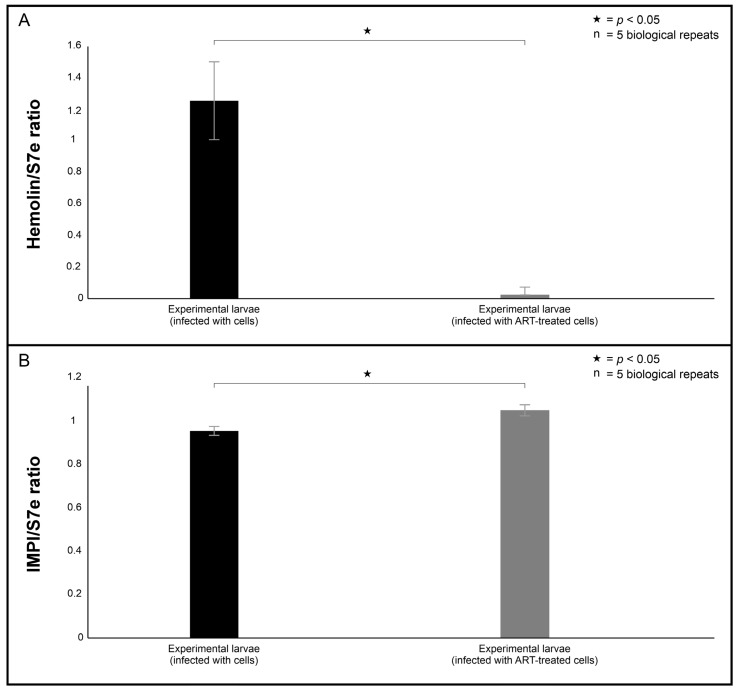
Characterisation of the larval immune response to infection with artemisinin (ART)-treated cells when compared to non-treated cells. The collated image shows the changes in the steady-state mRNA levels of hemolin (**A**) and IMPI (**B**) relative to the levels of s7e ribosomal gene, which was used as the internal housekeeping control. “n” = number of biological repeats.

To help explain the haemocyte phagocytosis data, the changes in the larval mRNA levels of hemolin and IMPI were analysed 24 h post-infection (Figure 8). These two markers were selected because they can offer insight into the insect’s immunological response. This is because hemolin is a pattern recognition protein from the immunoglobulin superfamily, which is typically upregulated during pathogen recognition and immune stimulation [21], while IMPI serves as a protective antiprotease that neutralises the pathogen-secreted metalloproteases [22], which are important fungal virulence factors. The infection of the larvae with 0.1 mM ART-treated cells provoked a weak immune response (*p* = 0.043) characterised by low expression of hemolin compared to larvae infected with 0 mM ART-treated cells (Figure 8A). On the other hand, the larvae maintained comparable levels (*p* = 0.025) of IMPI in both groups, i.e., larvae infected 0.1 mM ART-treated cells and 0 mM ART-treated cells (Figure 8B). 

**Figure 9:**
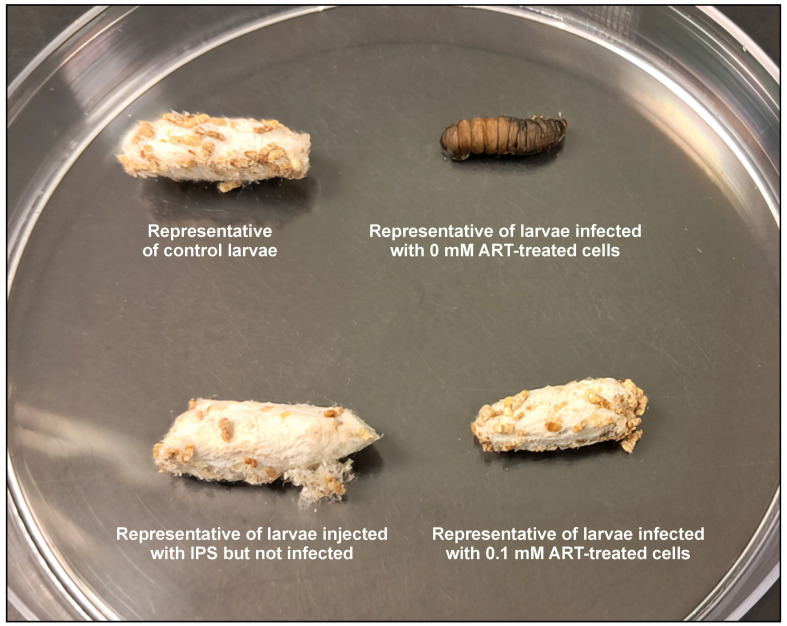
The image depicts the fate of the representative larvae, per experimental group. The results were recorded after all the larvae of the control group had formed cocoons.

**Figure 10:**
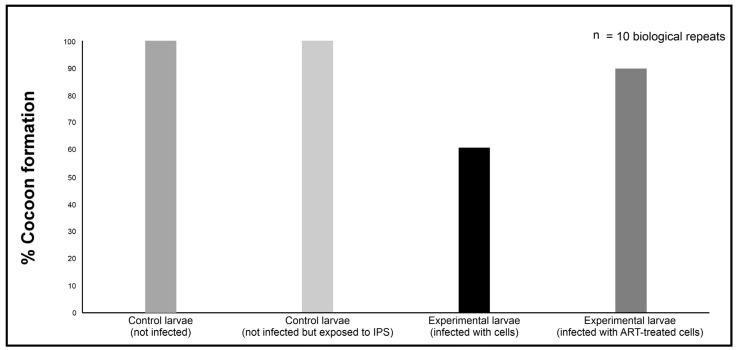
The quantification of the *Galleria mellonella* larvae’s ability to complete their life cycle and form cocoons following infection with the artemisinin (ART)-treated cryptococcal cells relative to the non-treated cells. If a larva could not form a cocoon, it implied it succumbed to the infection. Thus, cocoon formation was considered an indicator of survival, indicating that ART treatment rescued the larvae from succumbing to infection. “n” = number of biological repeats.

The latter may be critical to resolving a potential resurgence in fungal growth. It was noted that long-term, over the duration of the study, the larvae infected with 0 mM ART-treated cells could not complete their life cycle by forming cocoons; thus, they could not survive the infection (Figure 9 and Figure 10). Contrary to this, more of the larvae infected with 0.1 mM ART-treated cells could form cocoons. Importantly, when quantified, it was determined that the treatment rescued 75% of the infected larvae infected with 0.1 ART-treated cells.

## 3. Discussion

The presented study assessed the antifungal potential of ART through the use of in vitro assays that were complemented with the *Galleria* infection model. It was determined that ART could directly inhibit the growth of cryptococcal cells. Our data is in line with other studies documenting the antimicrobial activity of ART and its derivatives. Broadly, these studies highlight that the utility of ART and its derivatives may extend beyond their antifungal activity [14] to include serving as a potentiator of existing drugs, such as synergising with amphotericin B [15], and a modulator of virulence traits via disrupting biofilm development [16]. 

**Figure 11:**
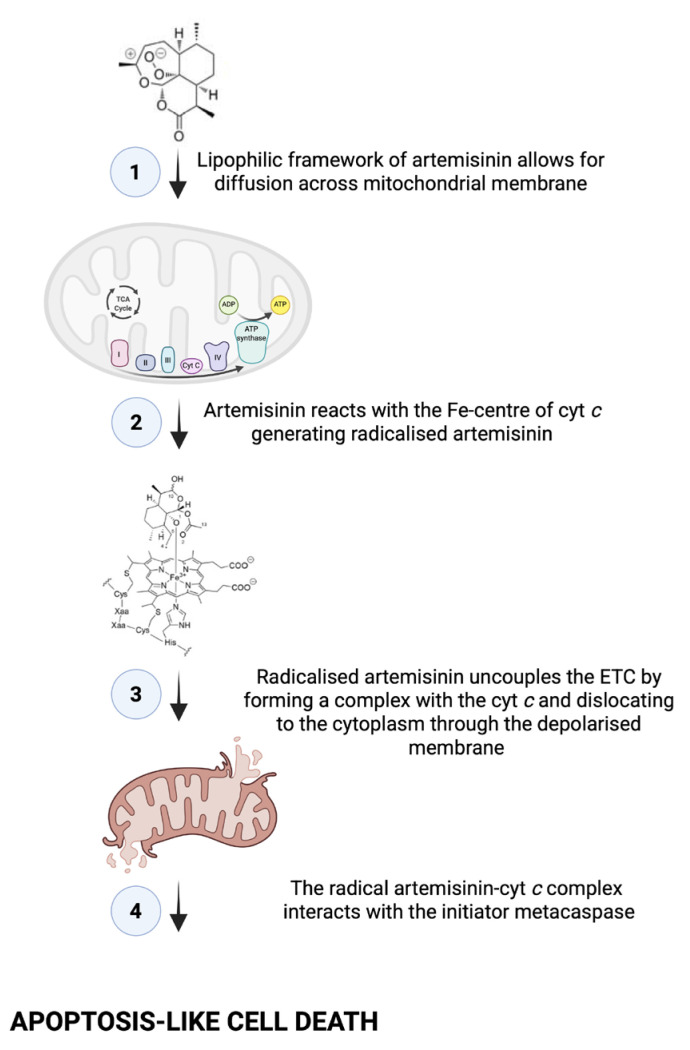
The proposed mechanism through which artemisinin (ART) induces death in treated cells. In the mechanism, the uncoupled cyt *c* is released from dysfunctional mitochondrial membranes to localise in the cytoplasm, where it triggers an apoptotic pathway that is dependent on the activity of caspase-3-like protease. The image was generated using bioRender.com.

When considering all our mitochondrial assay results (Figure 11), we rationalised that the treatment induced an apoptosis-like event, as *C. neoformans* does not possess true caspases [23]. This is because in mammalian cells, when cells undergo apoptosis, cyt *c* is released from the mitochondria and accumulates in the cytoplasm [24]. The event may be triggered by ART reacting with the Fe-centre of cyt *c* to form a monoradical ART-cyt *c*, as previously proposed by Laleve et al. [25], resulting in the cyt *c* traversing through the mitochondrial membrane that has now lost selective permeability to localise in the cytoplasm. There, the cyt *c* may bind to the initiator metacaspase, central to inducing apoptosis-like cell death in *C. neoformans* [26]. Furthermore, the dislocation of cyt *c* also suggests the electron transport chain (ETC) was uncoupled. 

As the ETC is highly regulated because of its involvement in energy production [27], the dislocation of cyt *c* implies that oxygen will become partly reduced, resulting in uncontrolled ROS production that may target cellular structures, as seen in Figure 4A. This deduction aligns with studies by Ogundeji et al. [28], which describes cryptococcal cellular damage following antifungal exposure. When taken together, these results again point to the ill fate of cells following treatment, as *C. neoformans* is a strict aerobe and cannot switch to a glycolytic fermentative pathway when its mitochondria are impaired.

**Figure 12:**
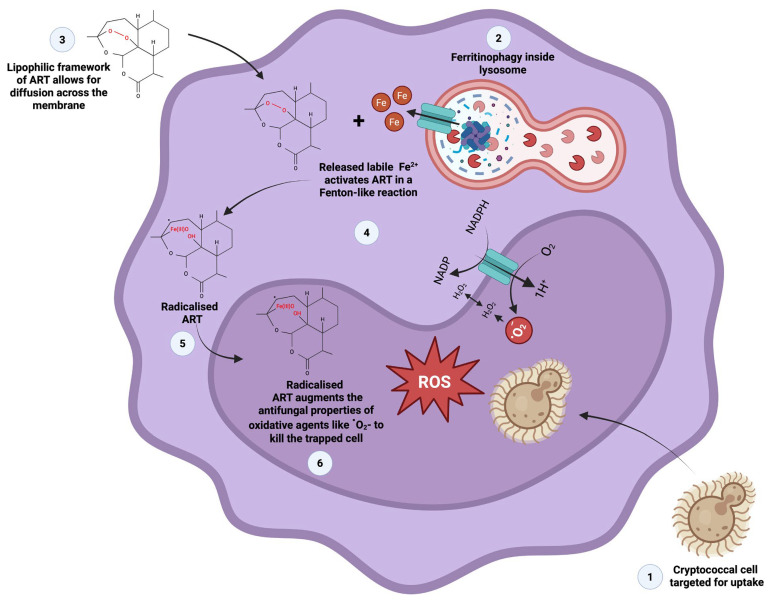
The proposed mechanism through which artemisinin (ART) achieves the killing of cryptococcal cells trapped inside macrophages or larval haemocytes. The image was generated using bioRender.com.

Cryptococcal cells have a particular preference to localise in the brain, where their presence results in meningitis development. Ma et al. [29] proposed vomocytosis as one of the processes that allow cells to reach the brain. In the process, cells manipulate phagocytic cells like macrophages in a Trojan horse-like manner to disseminate in the body, while haematogenously disseminating cells may resist phagocytosis by deploying the polysaccharide capsule to impede phagocytic uptake [18,30], including increasing the size of the capsule when cells enlarge to form titan cells [31,32]. The obtained haemocyte phagocytosis results suggest that phagocytic cells may respond to ART-treated cells across different host organisms in a similar manner. To this end, we theorise that the lipophilic framework of ART allows for this drug to permeate across the membrane into the cytoplasm of macrophages and haemocytes (Figure 12). 

Therein, this compound can be radicalised by free labile iron released from ferritin, in a Fenton-like reaction. The accumulation of the radicalised ART augments the effect of oxidative agents, like reactive oxygen species, which would in turn impose oxidative damage on internalised cryptococcal cells. As established in Figure 2, cryptococcal cell walls are susceptible to ART exposure. 

The gene expression results provided some insight into the insect antimicrobial response through the regulation of hemolin and IMPI towards both infection and ART exposure. Interestingly, the hemolin mRNA levels were lower in larvae infected with 0.1 mM ART-treated cells compared to the larvae infected with 0 mM ART-treated cells. Its reduced expression may reflect a diminished immune activation due to ART exposure, attenuating the immunostimulatory potential of treated cells, thereby lessening the need for host immune escalation. In contrast, IMPI mRNA levels remained comparable between the larvae infected with 0.1 mM ART-treated cells and the larvae infected with 0 mM ART-treated cells. In the context of haematogenously disseminating cryptococcal cells, cells secrete metalloproteases in order to traverse membrane barriers and enter organ systems [33]. Therefore, it could be reasoned that the comparable levels may suggest the immune system remains primed in case the infecting cells persist and secrete metalloproteases in the *Galleria*. Consistent with these immune results, the data revealed that treatment rescued 75% of the larvae infected with 0.1 ART-treated cells. Our findings are in line with other studies showing that antifungal exposure also enhanced larval survival [34].

## 4. Materials and Methods

### 4.1. Cells and the Organism Used in the Study

The cryptococcal laboratory reference strain, *C. neoformans* H99, which is kept as a culture at the University of the Free State, South Africa, was used in this study. This strain was cultured on yeast-malt extract agar (3 g/L yeast extract, 3 g/L malt extract, 5 g/L peptone, 10 g/L glucose, 16 g/L agar (Merck, Modderfontein, South Africa). The plates were incubated for 48 h at 30 °C. Five colonies were scooped from a plate and inoculated into 50 mL of yeast peptone dextrose medium (YPD, 10 g/L yeast extract, 20 g/L peptone, 20 g/L D-glucose; Merck, Modderfontein, South Africa). This culture was incubated for 18 h at 30 °C while shaking at 160 rpm. After 18 h, the cells were washed twice with PBS and standardised to 1 × 10^5^ cells per millilitre in 50 mL of fresh YPD broth. The prepared culture was grown for 24 h before the cells were standardised, likewise in YPD or Dulbecco’s Modified Eagle’s high glucose Medium (DMEM; Merck, Modderfontein, South Africa).

The murine macrophage cell line, RAW 264.7, which is kept as a culture at the University of the Free State, South Africa, was used in this study. The macrophages were cultivated in a tissue flask containing 10 mL of DMEM. The medium was supplemented with 10% (*v*/*v*) foetal bovine serum (Thermo Fisher Scientific, Johannesburg, South Africa), an antibiotic cocktail of penicillin (20 U/mL; Merck, Modderfontein, South Africa) and streptomycin (20 U/mL; Merck, Modderfontein, South Africa), as well as gentamycin (1%, Merck, Modderfontein, South Africa) until 80% confluency was reached. The viability of the cells was determined to be above 90% using the trypan blue stain (Merck, Modderfontein, South Africa). Using a haemocytometer (Marienfield Superior, Lauda-Königshofen Germany), the concentration of cells was adjusted to 1 × 10^5^ cells/mL. 

The *Galleria mellonella* larvae were reared on an artificial diet (400 g wheat bran, 100 g oat bran, 200 g nutty wheat, 200 g milk powder, 100 g instant yeast, 300 mL honey and 400 mL glycerol). These larvae were kept in glass jars at 28 °C in the dark until they reached the last instar stage. At this stage, their weight ranged between 150 mg and 200 mg. Ten larvae per experiment group were set aside and inspected for mobility and melanisation to avoid the use of animals that displayed signs of illness. Also, the selected larvae were surface sterilised using 70% ethanol (Merck, Modderfontein, South Africa) before use.

### 4.2. Preparation of Drugs

A standard powder of ART (Inqaba Biotechnical Industries. Pretoria, South Africa) and artesunate (ARS; Inqaba Biotechnical Industries. Pretoria, South Africa) were used in this study. A 2 mM stock solution of each drug was prepared in dimethylformamide (DMF; Sigma-Aldrich, Modderfontein, South Africa). These solutions were further diluted using YPD media, such that the final concentration of DMF was below 1%. These compounds were then tested at final concentrations of 0.001, 0.01 and 0.1 mM. 

### 4.3. In Vitro Susceptibility Testing Assay

The in vitro susceptibility of cryptococcal cells was determined using the EUCAST microbroth dilution protocol [35]. In brief, a 100 µL suspension of standardised cryptococcal cells (2 × 10^5^ cell/mL) was dispensed into wells of a 96-well plate. To the same wells, 100 µL of the drugs (ART and ARS) were separately dispensed into appropriate wells to reach a final test concentration of 0 mM, 0.001 mM, 0.01 mM and 0.1 mM. These plates were incubated for 48 h at 37 °C. After this incubation period, the optical density (OD) of each well was read at 595 nm using a Biochrom EZ Read 800 Research plate reader (Biochrom, Cambridge, UK). The half maximal inhibitory concentration (IC_50_) was determined by measuring the concentration that ART and ARS were required to respectively reduce the growth of treated cells by 50% relative to non-treated cells.

### 4.4. Electron Microscopy

Cryptococcal-treated cells, i.e., 0 mM ART and 0.1 mM ART, were prepared as stated in Section 4.3 and were subsequently used for electron microscopy examination. The scanning electron microscopy was based on the modified protocol of Swart et al. [36]. In brief, cells were fixed with equal amounts of buffered 3% (*v*/*v*; 0.1 mol/L) glutardialdehyde (Merck, Modderfontein, South Africa). The suspension was then rinsed with the same buffer to remove excess aldehyde fixative, and post-fixation was performed with 1% (*m*/*v*) buffered osmium tetraoxide (Merck, Modderfontein, South Africa). The suspension was washed to remove excess osmium tetraoxide. Dehydration was performed using a graded ethanol (Merck, Modderfontein, South Africa) sequence of 50, 70, 95 and 100% followed by drying using a critical point dryer. The dried material was mounted on aluminium SEM stubs and sputter-coated with gold (Merck). The samples were then examined with a scanning electron microscope (Shimadzu 1802 SSX-550 Superscan; Shimadzu. Tokyo, Japan) and photographed as digital images.

The transmission electron microscopy was based on the modified protocol of Swart et al. [36]. In brief, the fixed cells were embedded with epoxy resin in graded steps and allowed to polymerise for 8 h at 70 °C. An LKB III Ultratome was used to cut thin sections with glass knives. Sections were stained with uranyl acetate (Merck, Modderfontein, South Africa) and lead citrate (Merck, Modderfontein, South Africa). The prepared sections were examined with a CM 100 Philips transmission electron microscope (Philips/FEI Corporation, Eindhoven, The Netherlands) and photographed as digital images. To quantify the observed ultrastructural changes between the 0 mM and 0.1 mM experimental conditions, in the form of indentations, several images obtained from the different prepared sections depicting 100 cells were randomly selected. Thereafter, the % indentation was determined for each experimental condition.

### 4.5. Mode of Action: The Effect of ART on the Health of Treated Cells

Mitochondrial dysfunction offers essential insights into mitochondrial health, and by extension, cell vitality. To evaluate these aspects following ART treatment on cryptococcal cells, several assays were performed.

#### 4.5.1. Effect on the Production of Reactive Species (ROS)

The cells were handled as mentioned in Section 4.3, although the cells were treated with 0 mM ART and 0.1 mM ART for 6 h at 30 °C. The micro-well contents were aspirated and dispensed to corresponding wells in a black microtiter plate. Thereafter, 10 µL of 2’,7-dichlorofluorescein diacetate (Sigma-Aldrich, Modderfontein, South Africa) was reacted with 90 µL of the aspirated cell suspension. The plates were incubated at room temperature for 30 min in the dark. Induced fluorescence was measured at excitation of λex = 485 and emission of λem = 535 nm using a Thermo Scientific Fluoroskan microplate fluorometer(Thermo Scientific, Waltham, MA, USA).

#### 4.5.2. Effect on the Electron Transport Chain

To assess if treatment induced the uncoupling of the electron transport chain by releasing cytochrome (cyt *c*) from the mitochondria, a modified protocol by Choi and Lee [37] was followed. In brief, cells were handled as mentioned in Section 4.3, although the cells were treated with 0 mM ART, 0.1 mM ART or 50 mM CCCP (positive control) in 50 mL of YPD media for 6 h at 30 °C. Following treatment, cells were separated from the media by centrifugation and transferred to a 2 mL plastic tube that contained 1 mL of a buffer solution (50 mM Tris; Sigma-Aldrich, Modderfontein, South Africa), 2 mM EDTA (Sigma-Aldrich, Modderfontein, South Africa) and supplemented with 1 mM cOmplete™ protease inhibitor cocktail (Roche, Midrand, South Africa). The contents were prepared for homogenised with 0.5 mm diameter zirconium glass beads (Sigma-Aldrich, Modderfontein, South Africa). The plastic tube was placed on a BeadBug microtube homogeniser (Sigma-Aldrich, Modderfontein, South Africa) to achieve mechanical rupture. After homogenisation, 1 mL of 2% glucose (Merck) was added to the plastic tube. The tube was then centrifuged for 10 min at 2000× *g*, and the supernatant, representing cytoplasm with the dislodged cyt *c*, was aspirated, and a 100 µL suspension was dispensed into wells of a microtiter plate. The well contents were reacted with 100 µL of 500 mg/mL ascorbic acid (Sigma-Aldrich, Modderfontein, South Africa) for 5 min at room temperature. The absorbance of each plate was read at 558 nm using a Biochrom plate reader.

#### 4.5.3. Effect on Cell Membrane Integrity

The cells were handled as mentioned in Section 4.3, although the cells were treated with 0 mM ART or 0.1 mM ART for 6 h at 30 °C. The micro-well contents were aspirated and dispensed to corresponding wells in a black microtiter plate. Changes in the mitochondrial potential were measured by staining cells with the 5,5′,6,6′-tetrachloro-1,1′,3,3′-tetraethylbenzimidazolylcarbocyanine iodide dye (JC-10; Thermo Fisher Scientific, Johannesburg, South Africa). The plate was incubated for 30 min at 37 °C with 5% CO_2_. Following incubation, 50 µL of assay buffer was added to the wells, where a ratio of the fluorescence intensity (λex = 492/λem = 538 nm) and (λex = 538/λem = 590 nm) between the healthy and unhealthy cells was measured. 

The above assay was complemented by assessing whether ART induces loss of membrane’s selective permeability. The cells were handled as mentioned in Section 4.3, although the cells were treated with 0 mM ART or 0.1 mM ART for 6 h at 30 °C. The micro-well contents were aspirated and dispensed to corresponding wells in a black microtiter plate.

Loss of selective permeability was assessed by adding 1 µL of the propidium iodide exclusion stain (PI; Thermo Fisher Scientific, Johannesburg, South Africa) to the wells. The plate was incubated for 1 h at room temperature. Following incubation, fluorescence was measured at an excitation of λex = 485 nm and an emission of λem = 538 nm. 

#### 4.5.4. Effect on Apoptosis Induction

Caspase 3-like activity within 0 mM and 0.1 mM ART-treated cells was assessed using the Caspase-3 colorimetric activity assay kit (Merck, Modderfontein, South Africa) per the manufacturer’s instructions. The assay is based on monitoring the conversion of the tetrapeptide substrate DEVD-p-nitroanilide into the yellow-coloured p-nitroaniline (pNA). The micro-well contents containing the treated cells were aspirated and transferred to 1.5 mL plastic tubes. The cells were pelleted and resuspended in 50 µL of chilled 1x cell lysis buffer. The pelleted cells were incubated on ice for 10 min, and thereafter centrifuged for 10 min at 10,000× *g*. The supernatant was collected, and the protein concentration was normalised across the experimental conditions. The kinetic reaction was performed according to the manufacturer’s instructions, and the absorbance was measured at 405 nm using a Biochrom plate reader. A no-enzyme negative control was included to correct for nonspecific background.

### 4.6. Macrophage Toxicity and Phagocytosis Efficiency

To assess if ART was toxic to macrophages, its effect on macrophage metabolic activity was assessed using a 2,3-Bis(2-methoxy-4-nitro-5-sulfophenyl)-2H-tetrazolium-5-carboxanilide (XTT; Merck, Modderfontein, South Africa) assay based on a modified protocol by Ogundeji et al. [28]. In brief, 100 µL of the standardised macrophages (2 × 10^5^ cells/mL) were seeded in wells of a 96-well plate. To the same wells, 100 µL of ART was added to reach a final concentration of 0 (0x the IC_50_), 0.1 (1x the IC_50_), and 1 mM (10x the IC_50_). The plate was incubated in a 5% CO_2_ incubator (Thermo Scientific, Waltham, MA, USA) for 0, 6 or 24 h at 37 °C. At the end of the incubation period, the tetrazolium reaction was initiated in the presence of menadione (Merck, Modderfontein, South Africa) at 37 °C for 3 h in a 5% CO_2_ incubator. The absorbance of the wells was read at 492 nm following incubation.

The phagocytosis efficiency of macrophages was determined based on a modified protocol by Ogundeji et al. [28]. In brief, 100 µL of the standardised macrophages (2 × 10^5^ cells/mL) were seeded into adherent wells of a 96-well plate. Cryptococcal cells were prepared as stated in Section 4.3, although exposed to 0 and 0.1 mM of ART for 6 h. These cells were washed twice with PBS and resuspended in fresh DMEM media. These cryptococcal cells were added to the same adherent wells that contained macrophages to prepare an effector-to-target ratio of one fungus to one macrophage cell in each well. The plate was incubated in a 5% CO_2_ incubator for 2 or 6 h at 37 °C. At the end of the incubation period, the wells were washed twice with PBS to remove cryptococcal cells that were not internalised by macrophages. Thereafter, 200 μL of 0.1% Triton X-100 (Merck) was added to the same wells to lyse the macrophages. To ensure macrophages were lysed, the contents of each well were individually drawn in and out through a needle (26 gauge × 25 mm; Avacare, Kempton Park, South Africa) of a syringe 10 times. Following this, the contents of the wells were transferred to a 1.5 mL plastic tube and stained with 20 μL of trypan blue stain. A 10 μL suspension of the stained cells was aspirated, loaded into the Countess cell counting slide and inserted into an automated cell counter (Invitrogen, Carlsbad, CA, USA). 

### 4.7. Galleria Mellonella Infection Studies

Cryptococcal cells were prepared as stated in Section 4.3, although exposed to 0 mM ART and 0.1 mM of ART for 6 h before being used in the infection studies.

#### 4.7.1. Determining the Survival Rate Through Analysing the Effect of Infection on Cocoon Formation

The infection study was based on the modified protocol of Tsai et al. [38]. The selected healthy and surface-sterilised *Galleria mellonella* larvae were restrained gently and injected through the last left proleg with a 10 μL suspension of cryptococcal cells treated with 0.1 mM of ART (prepared as stated in Section 4.3), using a Hamilton syringe with a 26-gauge needle. Control groups received 10 μL of sterile insect physiological saline (IPS; 50 mM NaCl, 5 mM KCl, 100 mM Tris-HCl, pH 6.9; Merck, Modderfontein, South Africa) but were not infected, or 10 μL suspension of cryptococcal cells treated with 0 mM of ART. Additionally, a control group of larvae not infected with cryptococcal cells or injected with IPS was included for comparison purposes. All the groups were made of 10 larvae. After handling, the larvae were incubated in Petri dishes. The survival rate was determined by assessing the number of larvae that could transition and form cocoons and expressed as a percentage. This implied that the larvae that could not form cocoons succumbed to the infection, while those that could, the ART treatment contributed towards infection resolution. The larvae were monitored daily post-infection until all the larvae of the control group, i.e., those that received 10 μL of sterile insect physiological saline, formed cocoons, and then the study was terminated. To calculate the ability of ART to rescue larvae from succumbing to infection, the following formula was used:

$$\mathrm{Rescue}\;\mathrm{Rate}\;(\%)=(\frac{{\mathrm{Survival}}_{\mathrm{treated}}-{\mathrm{Survival}}_{\mathrm{infected}}}{{\mathrm{Survival}}_{\mathrm{uninfected}}-{\mathrm{Survival}}_{\mathrm{infected}}})\times 100$$

#### 4.7.2. Haemocyte Phagocytosis

Haemocyte phagocytosis was based on the protocol of Campbell et al. [39]. Additional larvae were prepared as mentioned in Section 4.7.1. For this assay, only the larvae that received 10 μL suspension of cryptococcal cells treated with 0 mM of ART, as well as 1 mM of ART, were considered. Twenty-four hours post-infection, 20 μL of haemolymph, containing haemocytes, was collected into ice-cold 1.5 mL tubes containing a few crystals of phenylthiourea (Merck, Modderfontein, South Africa) in IPS to prevent melanisation by puncturing the larval thorax. The haemolymph was transferred to black 96-well plates, and a pHrodo stain (Thermo Fisher Scientific, Johannesburg, South Africa) was added to the wells (1:2000; *v*/*v*) to measure haemocyte phagocytosis efficiency using a modified protocol based on the manufacturer’s instructions for using the pHrodo stain. The plate was incubated for 1 h at 37 °C. Following incubation, fluorescence was measured at an excitation of λex = 485 nm and an emission of λem = 538 nm to assess phagocytosis.

#### 4.7.3. Determining the Effect of Infection on the Larval mRNA Levels of Hemolin and IMPI 

Additional larvae were prepared as mentioned in Section 4.7.1. For this assay, only the larvae that received 10 μL suspension of cryptococcal cells treated with 0 mM of ART, as well as 1 mM of ART, were considered. Twenty-four hours post-infection, the larvae were flash-frozen using liquid nitrogen and crushed using a pestle and mortar. The E.Z.N.A. HP Total RNA Kit was used to extract total RNA according to the manufacturer’s protocol (Whitehead Scientific, Cape Town, South Africa). The purified RNA was used in gel electrophoresis (1% (*w*/*v*) agarose bleach gel; 100 V, 45 min) and visualised under a transilluminator. The RNA was also quantified using the Qubit™ RNA IQ Assay kit (Thermo Fisher Scientific, Johannesburg, South Africa). RT-qPCR was performed using the qScript One-Step SYBR Green qRT-PCR kit. Thermal cycling conditions were as follows: initial hold at 50 °C for 10 min; initial denaturation at 95 °C for 5 min; 40 cycles of 95 °C for 5 s and 60 °C for 30 s. Two immunological markers, hemolin and IMPI, were assessed, where changes in steady-state mRNA levels were quantified relative to the levels of s7e as the internal housekeeping control. All reactions were run in triplicate, and non-template controls were included.

### 4.8. Statistical Analyses

Unless stated otherwise, for each study, five independent biological experiments were performed. GraphPad Prism 8.3.1 was used to calculate mean values and the standard deviation of the means. The same programme was used to perform the multiple comparison test using Tukey as an option. A *p*-value of less than or equal to 0.05 was considered significant.

## 5. Conclusions

In conclusion, our in vitro results highlight the potential utility of ART as an anti-*Cryptococcus* antifungal. The use of *Galleria* provided a valuable bridge between the in vitro findings and animal infection studies, by providing valuable insight into how ART may influence both pathogen viability and host immunity. To validate the effectiveness of ART, it is essential that a future study tests it using a laboratory murine model as the next step in the translational programme to help justify and de-risk potential clinical studies.

## Data Availability

All the generated data in the study are presented in the current paper and are available from the corresponding author upon reasonable request.
